# Immunohistochemistry localises myosin-7a to cochlear efferent boutons

**DOI:** 10.12688/wellcomeopenres.17428.2

**Published:** 2022-02-22

**Authors:** Piotr Sirko, Andrei S. Kozlov

**Affiliations:** 1 Laboratory of Auditory Neuroscience and Biophysics, Department of Bioengineering, Imperial College London, London, SW7 2AZ, UK

**Keywords:** Myosin 7a, medial olivocochlear fibres, hair cells, Usher syndrome

## Abstract

**Background: **Myosin 7a is an actin-binding motor protein involved in the formation of hair-cell stereocilia both in the cochlea and in the vestibular system. Mutations in myosin 7a are linked to congenital hearing loss and are present in 50% of Type-1 Usher syndrome patients who suffer from progressive hearing loss and vestibular system dysfunction.

**Methods: **Myosin 7a is often used to visualise sensory hair cells due to its well characterised and localised expression profile. We thus conducted myosin-7a immunostaining across all three turns of the adult rat organ of Corti to visualise hair cells.

**Results: **As expected, we observed myosin 7a staining in both inner and outer hair cells. Unexpectedly, we also observed strong myosin 7a staining in the medial olivocochlear efferent synaptic boutons contacting the outer hair cells. Efferent bouton myosin-7a staining was present across all three turns of the cochlea. We verified this localisation by co-staining with a known efferent bouton marker, the vesicular acetylcholine transporter.

**Conclusions: **In addition to its role in stereocilia formation and maintenance, myosin 7a or certain myosin-7a expression variants might play a role in efferent synaptic transmission in the cochlea and thus ultimately influence cochlear gain regulation. Our immunohistochemistry results should be validated with other methods to confirm these serendipitous findings.

## Introduction

Usher syndrome is an autosomal recessive disorder which affects hearing, vision and balance in approximately 4 to 17 per 100,000 people ^1, 2^. About 50% of hereditary hearing and vision loss cases have been linked to Usher syndrome ^3^. Although the mechanisms underlying Usher syndrome are not entirely clear, many of the mutations that cause it affect proteins expressed in sensory hair cells. Studies on these proteins show that most of them are involved in the formation or maintenance of hair-cell stereocilia, which play a key role in the transmission of acoustic or vestibular stimuli ^4^.

One of such proteins is myosin-7a, an actin-binding motor protein. In hair cells myosin-7a has been linked to the transport of other proteins along the actin filaments inside stereocilia and the maintenance of stereocilia structure ^5, 6^. Thus, it plays an important role in stereocilia formation and maintenance. Mutations in myosin-7a have been linked to the most severe Usher syndrome, Type 1, and account for 50% of Usher Type 1 cases and 21% of all Usher syndrome cases ^7– 11^. Myosin-7a mutations have also been linked to non-syndromic deafness ^12, 13^.

In addition to its presence in stereocilia, myosin-7a is present throughout the hair cell body and is not significantly expressed in the non-sensory cells of the organ of Corti. Given its localisation in hair cells and good availability of high-quality antibodies, myosin-7a is often used to stain hair cells selectively in cochlear immunohistochemistry investigations ^14^.

In the mammalian cochlea, two types of hair cells are present. Inner hair cells convert sound stimuli into electrical signals which can be transmitted to higher auditory processing regions. Whereas outer hair cells appear to be mostly involved in the enhancement of sound-induced vibrations in the cochlea, and thus increase the “gain” of the signal reaching the inner hair cells ^15– 17^.

Studies indicate that the level of “gain” increase by the outer hair cells can be adjusted by cholinergic efferent fibres which originate in the brainstem and directly synapse onto the base of outer hair cells. Regulation of the “gain” by these medial olivocochlear fibres might be key to our ability to understand complex sounds such as speech in noisy environments and is thought to exert a protective effect when the ear is exposed to louder sounds ^18– 20^.

**Figure 1.**
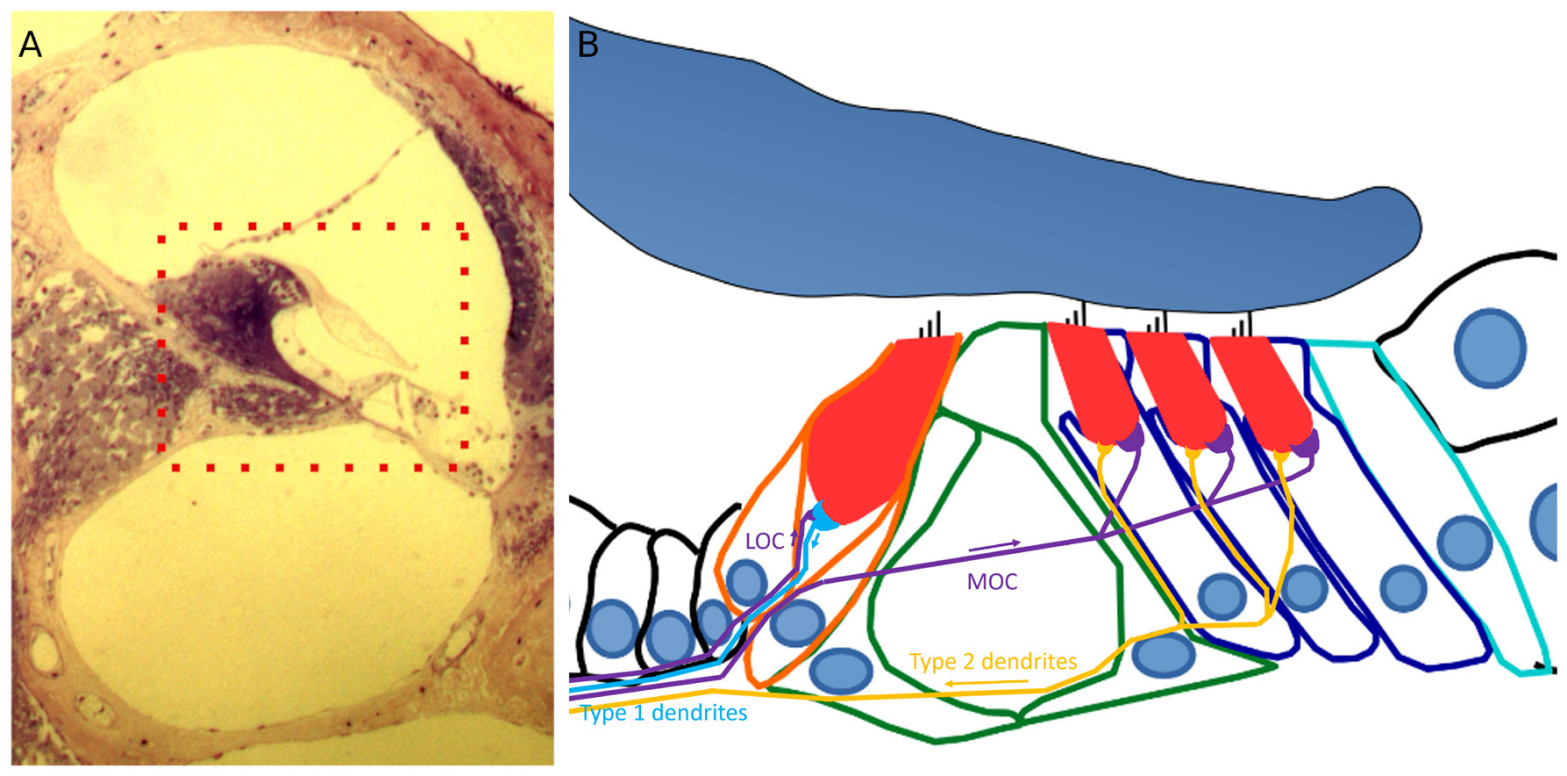
Cross-section of the cochlea and the organ of Corti. **A**. A section through the cochlea, rectangle depicts the location of the organ of Corti. **B**. A diagram representation showing a cross-section through the organ of Corti. Hair cells (red) can be subdivided into outer hair cells organised into three rows and inner hair cells. The medial and lateral efferent boutons and fibres (MOC and LOC) are depicted in purple. The afferent type 1 dendrites are depicted in blue and type 2 dendrites are shown in yellow. Cochlear section was a gift of Prof. Andrew Forge.

A similar protective role is ascribed to the cholinergic lateral olivocochlear fibres which synapse onto the type 1 afferent neurons carrying sound information near the base of the inner hair cells ^18^ ( Figure 1). During our investigation of the adult rat cochlea we not only observed myosin-7a staining in stereocilia and hair cell bodies, but also found strong myosin-7a staining in the medial olivocochlear boutons synapsing onto outer hair cells. This suggests that in addition to its role in hair cells, myosin-7a might play a role in cochlear gain regulation.

Our findings also suggest that myosin-7a mutations might contribute to hearing loss in Usher syndrome and nonsyndromic deafness patients by affecting efferent feedback function.

## Methods

The results described in this study were obtained as part of our research on the mechanisms of blast damage. All animal experiments were conducted under the Home Office project license P5B192285, were approved by the Imperial College AWERB Committee, and were in accordance with the UK Animals (Scientific Procedures) Act (1986). Some rats were subjected to a mild form of blast injury 3 months before cochlea isolation. The blast procedure was carried out using a compressed-gas driven shock tube of the Centre for Blast Injury Studies at Imperial College London. The configuration yielded a Friedlander pressure waveform with peak pressure of 230 kPa that simulates open-field detonations. We did not observe any immunostaining pattern differences between blasted and sham rats in the results described in this study. Hence we do not further distinguish between these two groups of rats in this article.

Cochleas were isolated from adult Sprague-Daley male rats (>400g) which corresponds to an age of 10 weeks and older. Rats were killed in accordance with UK Home Office Schedule 1 guidelines and decapitated. Intact cochleas were separated from the temporal bone, fixed and stored in 4% PFA at 4 °C for at least 24 hours. Data in this study were gathered from 11 cochleas obtained from 7 rats. Where possible we aimed to replicate data in 3 separately stained cochleas from 3 animals. We did not need to exclude any cochleas from our analysis.

Cochleas were washed 3 times for 5 minutes each in PBS to remove the PFA and transferred to a fresh batch of PBS. Excess tissue was removed and the bone covering the organ of Corti was carefully removed with tweezers. After exposing the organ of Corti, the tectorial membrane was peeled away with tweezers from the middle and base turns. To block nonspecific antigens, cochleas were incubated in blocking solution consisting of PBS, 0.1% Triton X-100 and 5% Normal Goat Serum for one hour at room temperature whilst placed on a laboratory rocker. After blocking, cochleas were moved to a fresh batch of blocking solution with primary antibodies and left to incubate and rock for 4 hours at room temperature. Subsequently, cochleas were washed 3 times for 5 minutes each using PBS and transferred to blocking solution with added secondary antibodies and phalloidin for 3 hours, again whilst rocking at room temperature. Finally, cochleas were washed 3 times for 5 minutes using PBS and stored in PBS until imaging.

Table 1 lists the primary antibodies used and their concentrations. Secondary anti-rabbit, anti-chicken and anti-guinea pig antibodies (Invitrogen) conjugated to Alexa fluorophores (488, 546, 594, 633) were used at a final concentration of 1:300 as summarised in Table 2. Phalloidin conjugated to Alexa 405 at a final concentration of 1:300 was added during the secondary antibody incubation step to visualise actin-rich stereocilia (A30104: Alexa Fluor™ Plus 405 Phalloidin Invitrogen). We used either anti-rabbit antibodies conjugated to Alexa 488 or 594 to visualise myosin-7a. We also used an anti-guinea pig antibody conjugated to Alexa 546 to visualise vesicular acetylcholine transporter (VAChT) as well as an anti-chicken antibody conjugated to Alexa 633 to visualise Neurofilament-Heavy (NF-H). Artificial look up table colors were applied to the captured images to better visualise and contrast the staining patterns. The chosen colours do not necessarily reflect the wavelength at which fluorescence was recorded.

**Table 1.** Primary antibodies used in the study.

Antibody	Antigen	Host	Supplier	Dilution
PA1-936	Mouse myosin-7a	Rabbit polyclonal	Invitrogen	1:300
139 105	Rat VAChT	Guinea pig polyclonal	Synaptic Systems	1:300
AB5539	Bovine NF-H	Chicken polyclonal	Sigma-Aldrich	1:600

**Table 2.** Secondary antibodies used in the study.

Antibody	Antigen	Host	Supplier	Dye	Dilution
A11008	Rabbit IgG	Goat polyclonal	Invitrogen	Alexa Fluor 488	1:300
A11012	Rabbit IgG	Goat polyclonal	Invitrogen	Alexa Fluor 594	1:300
A21103	Chicken IgY	Goat polyclonal	Invitrogen	Alexa Fluor 633	1:300
A11074	Guinea pig IgG	Goat polyclonal	Invitrogen	Alexa Fluor 546	1:300

A Leica SP5 upright confocal microscope with two-photon imaging capabilities was used to image the exposed organs of Corti. Cochleas were superglued to the lids of 55-mm diameter cell culture Petri dishes prior to imaging to ensure the organ of Corti would be in an appropriate orientation and immersed in PBS.

The Argon 488, Diode 543, Diode 594 and Diode 633 were used for single-photon excitation of the Alexa dyes conjugated to the secondary antibodies and the Mai Tai eHP DeepSee 5332 laser set to a wavelength of 800 nm was used for two-photon excitation of the phalloidin-conjugated Alexa 405. A 25x/0.95NA water immersion objective (HCX IRAPO L25x/0.95 W) was used. Images were recorded in 12 bits at a resolution of 1024 x 1024 and further processed in ImageJ ( https://imagej.nih.gov/ij/)

## Results

**Figure 2.**
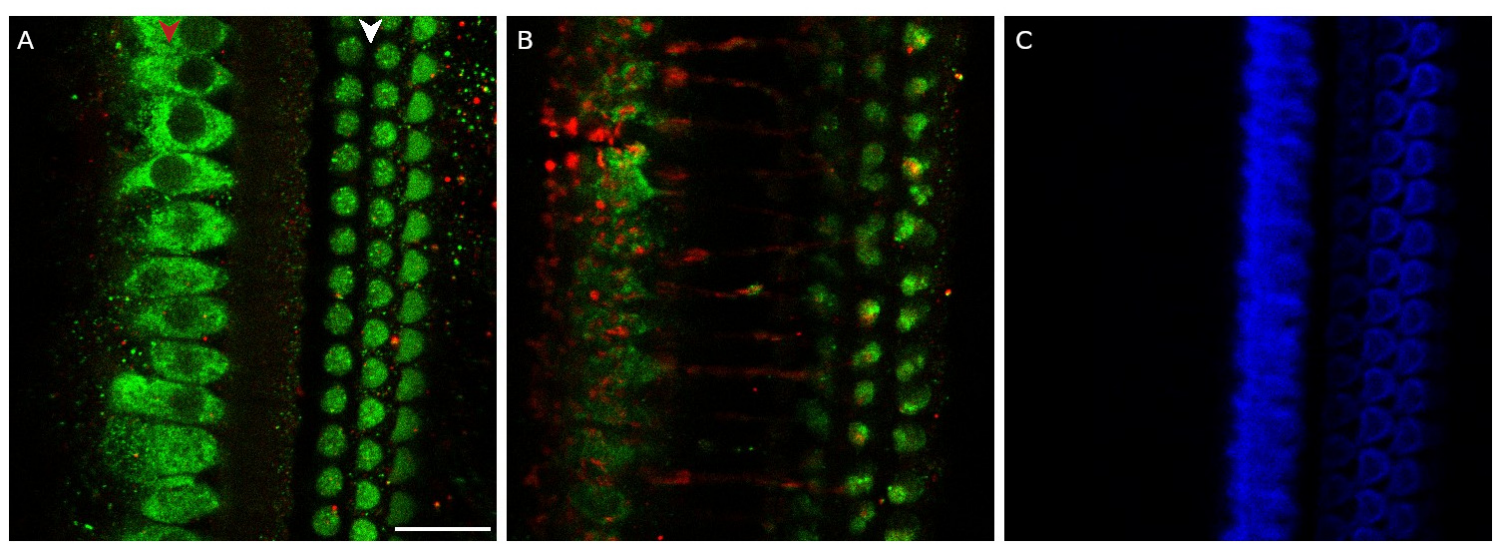
Myosin-7a staining of medial olivocochlear boutons in the basal turn of the rat organ of Corti. **A**. Composite picture showing myosin-7a (green) and NF-H (red) staining at the level of the top of outer hair cells and **B**. below the outer hair cells at the level of the medial olivocochlear boutons, which were located approximately 12 µm below the apical surface of the outer hair cells. **C**. Phalloidin (blue) staining of the same organ of Corti fragment showing the tops of the outer hair cells. Red arrowhead indicates the row of the inner hair cells, white arrowhead indicates the three rows of outer hair cells. Scale bar is 20 µm. Data was replicated in 3 cochleas from 3 rats.

**Figure 3.**
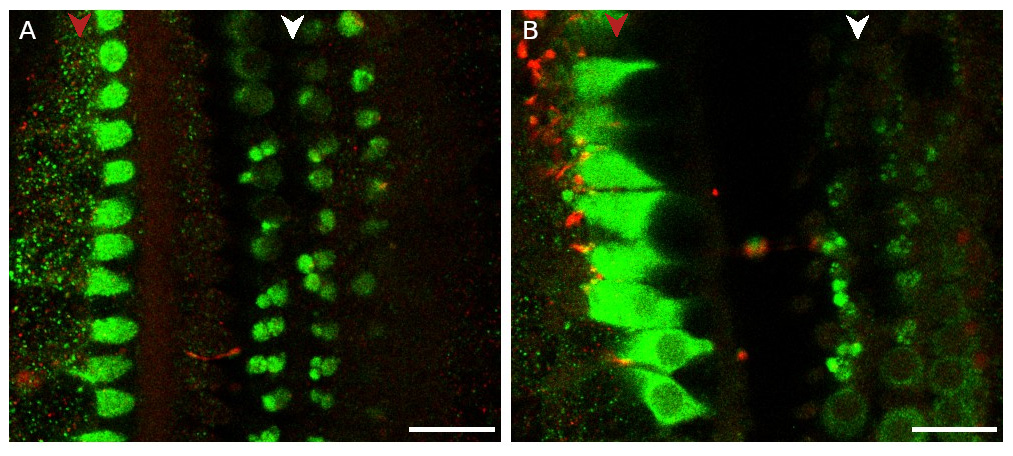
Myosin-7a stains medial olivocochlear boutons in the middle and apical turn of the rat organ of Corti. **A**. Medial olivocochlear boutons are visible below the outer hair cells in the mid and in **B**. the apical turn. (green – myosin-7a, red – NF-H). Red arrowhead indicates the row of the inner hair cells, white arrowhead indicates the three rows of outer hair cells. The medial olivocochlear boutons were located approximately 15 to 25 µm below the apical surface of the outer hair cells. Scale bars are 20 µm. Mid-turn data were replicated in 3 cochleas from 3 rats. Apical region data were replicated in 3 cochleas from 2 rats.

**Figure 4.**
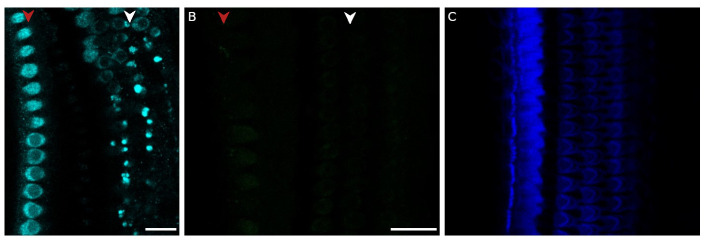
Changing the secondary antibody did not change the myosin-7a staining pattern. **A**. Myosin-7a staining (cyan) in the apical cochlear turn using an Alexa-594 anti-rabbit secondary antibody instead of Alexa-488 antirabbit (Note that the cyan colour does not correspond to the wavelength of the fluorescent signal). **B**. Negative control with only secondary Alexa 488 antibody (left) and phalloidin (right). Red arrowhead indicates the row of the inner hair cells, white arrowhead indicates the three rows of outer hair cells. Scale bars are 20 µm. Each control was conducted on a single cochlea, each from a different rat.

Cochleas were stained using the myosin-7a antibody and phalloidin to visualise hair cells and stereocilia, and the NF-H antibody was used to visualise afferent and efferent fibers. Myosin-7a antibodies in conjunction with secondary anti-rabbit Alexa 488 conjugated antibodies visualised inner and outer hair cells across all 3 cochlear turns in the rat, consistent with a number of previous investigations ^21– 23^ ( Figure 2 and Figure 3). In addition, in the outer hair cell region, high-intensity myosin-7a staining was visible manifesting as oval structures which appeared to be just below the outer hair cells. These oval structures were visible in all 3 turns of the rat organ of Corti with typically 1 to 3 of them apparent below each outer hair cell. As we did not find any equivalent finding published in the literature we investigated if this staining was due to nonspecific binding of the secondary antibodies. To try to exclude this possibility we used a different secondary anti-rabbit antibody conjugated to Alexa 594 instead of Alexa 488. The oval structures were still visible when using the Alexa-594 conjugated anti-rabbit antibody ( Figure 4). As an additional control, we also omitted the primary myosin-7a antibody. With no primary antibody present no oval structures were visible.

**Figure 5.**
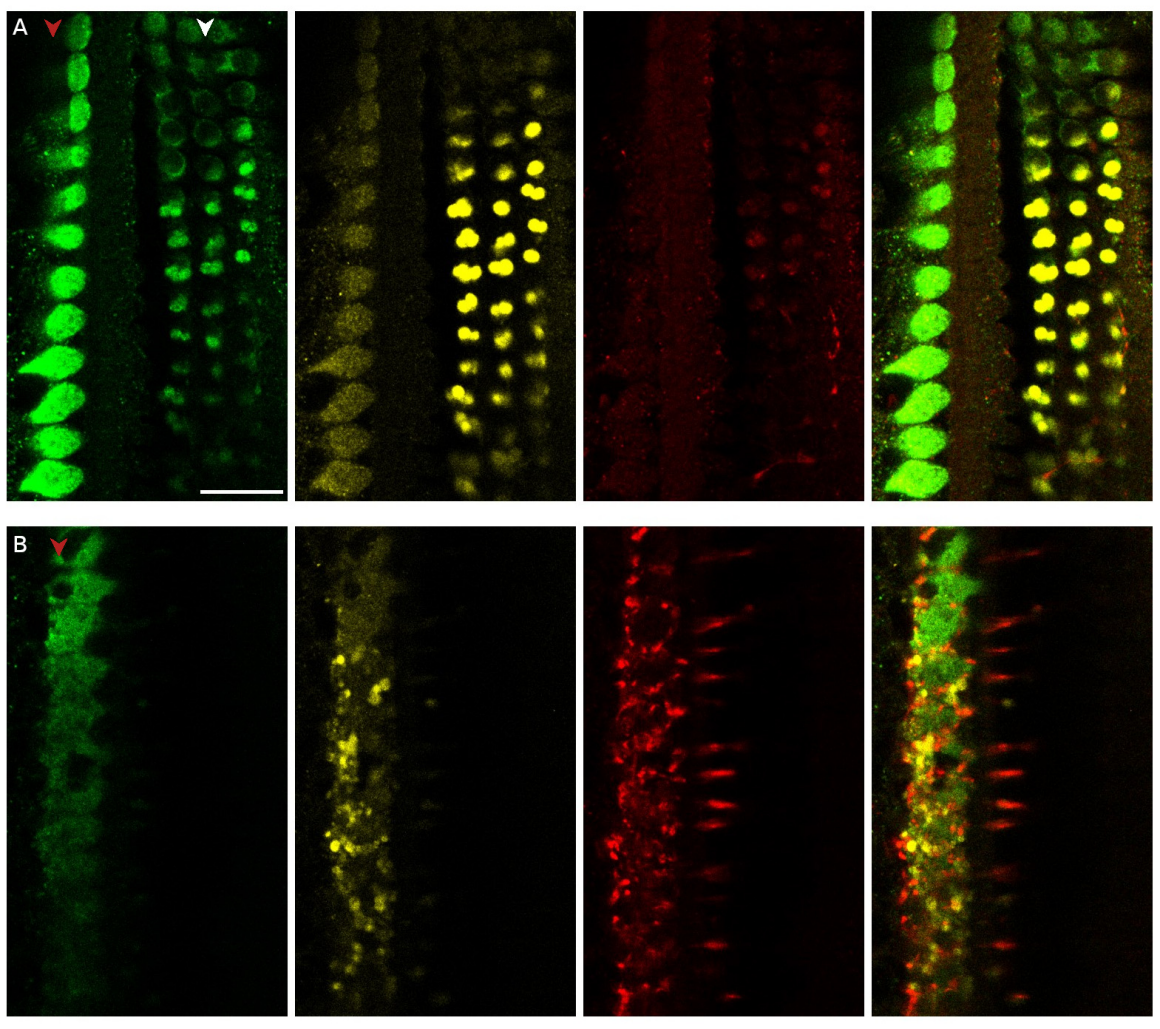
Myosin-7a staining visualises medial olivocochlear boutons but not lateral olivocochlear boutons. **A**. Myosin-7a (green), VAChT (yellow), NF-H (red) and composite at the plane of the medial olivocochlear boutons. B. Myosin-7a (green), VAChT (yellow), NF-H (red) and composite at the plane of the lateral olivocochlear boutons. Red arrowhead indicates the row of the inner hair cells, white arrowhead indicates the three rows of outer hair cells. The medial olivocochlear boutons were located approximately 12 µm below the apical surface of the outer hair cells. The lateral olivocochlear boutons were located approximately 20 µm below the apical surface of the inner hair cells. Scale bar is 20 µm. Data were replicated in 2 cochleas from 2 rats.

As the observed oval structures resembled medial olivocochlear boutons that synapse onto the outer hair cells, we co-stained with an antibody against the VAChT, which is used to visualise medial and lateral olivocochlear boutons. The oval structures visualised using the myosin-7a antibody co-stained with the VAChT. In addition, the VAChT antibody visualised the lateral olivocochlear boutons in the inner hair cell region, which were not visualised by myosin-7a staining ( Figure 5).

**Figure 6.**
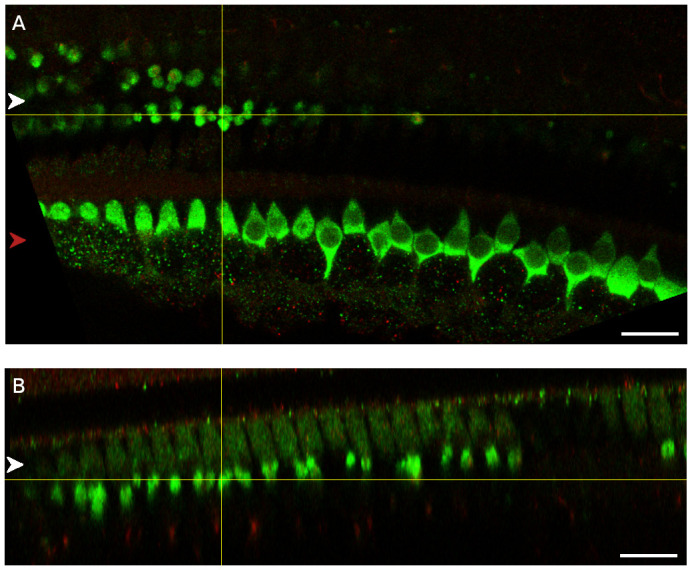
Strong myosin-7a staining is visible below the outer hair cells in the mid cochlear turn. **A**. Top down view showing myosin-7a (green) and NF-H (red) staining. The yellow x-axis represents the plane along which the orthogonal view shown in **B** was created. **B**. Orthogonal view showing a cross-section of the organ of Corti along the yellow x-axis shown in **A**. Red arrowhead indicates the row of the inner hair cells, white arrowhead indicates the three rows of outer hair cells or a single row in the orthogonal view. The medial olivocochlear boutons were located approximately 15 µm below the apical surface of the outer hair cells. Scale bar is 20 µm.

**Figure 7.**
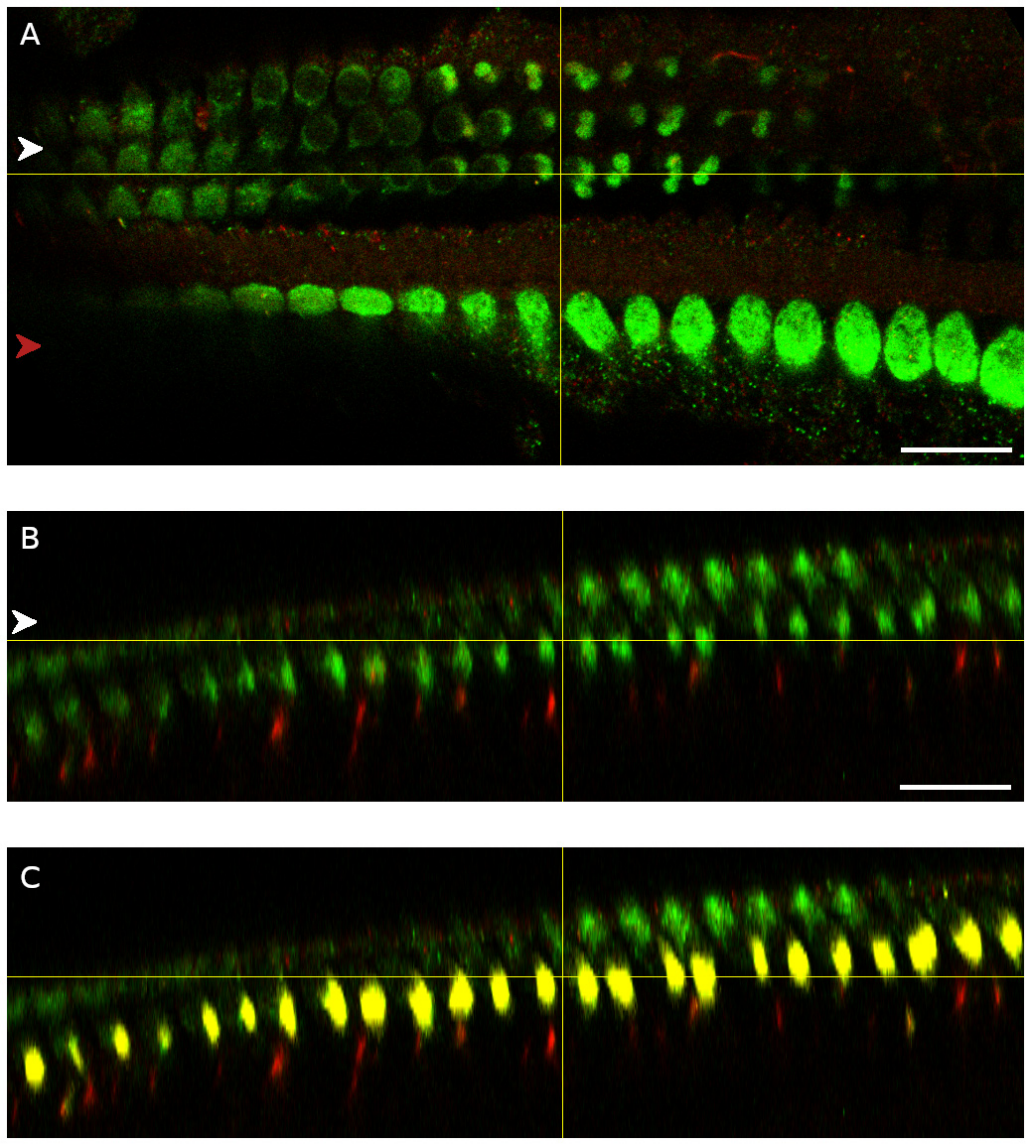
Strong myosin-7a and VAChT staining is visible below the outer hair cells in the basal cochlear turn. **A**. Top down view showing myosin-7a (green) and NF-H (red) staining. The yellow x-axis represents the plane along which the orthogonal views shown in **B** and **C** were created. **B**. Orthogonal view showing a cross-section of the organ of Corti along the yellow x-axis shown in **A**. **C**. Orthogonal view showing a cross-section of the organ of Corti along the yellow x-axis shown in **A** and VAChT staining (yellow). Red arrowhead indicates the row of the inner hair cells, white arrowhead indicates the three rows of outer hair cells or a single row in the orthogonal view. The medial olivocochlear boutons were located approximately 12 µm below the apical surface of the outer hair cells. Scale bars are 20 µm.

To further confirm the location of the myosin-7a staining in the outer hair cell region relative to hair cells and the VAChT we created orthogonal views showing a cross-section of the organ of Corti at locations in which the oval myosin-7a staining structures were observed ( Figure 6 and Figure 7). These show that the myosin-7a-rich oval structures are located towards the bottom of the outer hair cells, that the shape of their cross-section is not inconsistent with the shape of synaptic boutons and that their localisation corresponds with that of the VAChT. In addition, the myosin-7a-rich oval structures can be distinguished from the outer hair cells thanks to the higher fluorescent signal level.

## Discussion

Our results suggest that in addition to being present in hair cells, myosin-7a may also be present in the medial olivocochlear boutons contacting outer hair cells.

We have observed myosin-7a staining in the medial olivocochlear boutons across all three turns of the cochlea and verified that the staining pattern we observe is not due to unspecific staining related to the secondary antibody. We have also independently confirmed localisation to the medial olivocochlear boutons by using a known cochlear efferent bouton marker VAChT. In contrast to myosin-7a staining, VAChT staining also visualised the lateral olivocochlear boutons in the inner hair cell region, which is a further positive control validating that the myosin-7a staining in the medial olivocochlear boutons is specific. Future work should validate myosin-7a localisation to medial olivocochlear boutons in other species and using other experimental methods.

Several studies investigating myosin-7a expression in the cochlea have been conducted in the past and it is not clear why it has not been observed in medial olivocochlear boutons previously. One possibility is that the polyclonal antibody we have used to visualise myosin-7a recognises an epitope, which is present in myosin-7a isoforms specific to the medial olivocochlear boutons. We have determined that the antibody we have used was raised using an N-terminal antigenic region of myosin-7a, in contrast to other popular myosin-7a antibodies raised using antigenic regions in the C-terminal region of myosin-7a used in many other publications ^6, 14, 24^. It is not clear if all myosin-7a isoforms would have this same C-terminal region. The C-terminal region of myosins is documented to be variable, and differences might affect the subcellular distribution and function of different myosin isoforms ^25, 26^.

According to the website of the antibody’s manufacturer, this particular myosin-7a antibody has been used in the past to visualise myosin-7a as part of other published studies. These however have not involved staining of the adult rat cochlea and were mostly done on mice before the onset of hearing ^27– 29^. Therefore, if myosin-7a presence in medial olivocohlear boutons is related to maturation state or this particular epitope is present only in the rat, then no evidence of staining would have been present in these studies.

Although we have attempted to exclude nonspecific staining, it is worth noting that there is a possibility the myosin-7a antibody we used stains a different protein present in the medial olivocochlear boutons. However, as many of the antibodies which can be used to visualise the medial olivocochlear boutons, such as VAChT, also label the lateral olivocochlear boutons, it is interesting to note that the myosin-7a antibody stains for a protein that is only found in medial olivocochlear boutons. Therefore, even if a different protein than myosin-7a is responsible for the staining pattern we observed, it would be still advantageous to use it as a highly specific marker of medial olivocochlear boutons, as well as potentially important to determine its function.

If myosin-7a is indeed present in the medial olivocochlear boutons, it could hint at another important role this protein plays within the cochlea and possibly have consequences for our understanding of the mechanisms underlying Usher syndrome and congenital hearing loss.

## Data availability

### Underlying data

Dryad: Immunohistochemistry localises myosin-7a to cochlear efferent boutons, https://doi.org/10.5061/dryad.9s4mw6mhz ^30^

This project contains the following underlying data:

- A zipped file containing tiff files organised in folders based on which figure in this publication the data are associated with.

Data are available under the terms of the Creative Commons Zero "No rights reserved" data waiver (CC0 1.0 Public domain dedication).

### Reporting guidelines

Zenodo: ARRIVE checklist for ‘Immunohistochemistry localises myosin-7a to cochlear efferent boutons’, https://doi.org/10.5281/zenodo.5763739 ^31^

Data are available under the terms of the Creative Commons Attribution 4.0 International license (CC-BY 4.0).

## References

[1] Boughman JA Vernon M Shaver KA : Usher syndrome: definition and estimate of prevalence from two high-risk populations. *J Chronic Dis.* 1983;36(8):595–603. 10.1016/0021-9681(83)90147-9 6885960

[2] Kimberling WJ Hildebrand MS Shearer AE : Frequency of usher syndrome in two pediatric populations: Implications for genetic screening of deaf and hard of hearing children. *Genet Med.* 2010;12(8):512–6. 10.1097/GIM.0b013e3181e5afb8 20613545 PMC3131500

[3] Ben-Rebeh I Grati M Bonnet C : Genetic analysis of tunisian families with usher syndrome type 1: toward improving early molecular diagnosis. *Mol Vis.* 2016;22:827–35. 27440999 PMC4950652

[4] Cosgrove D Zallocchi M : Usher protein functions in hair cells and photoreceptors. *Int J Biochem Cell Biol.* 2014;46:80–9. 10.1016/j.biocel.2013.11.001 24239741 PMC3971483

[5] El-Amraoui A Petit C : Usher i syndrome: unravelling the mechanisms that underlie the cohesion of the growing hair bundle in inner ear sensory cells. *J Cell Sci.* 2005;118(Pt 20):4593–603. 10.1242/jcs.02636 16219682

[6] Morgan CP Krey JF Grati M : Pdzd7-myo7a complex identified in enriched stereocilia membranes. *eLife.* 2016;5:e18312. 10.7554/eLife.18312 27525485 PMC5005036

[7] Weil D Blanchard S Kaplan J : Defective myosin viia gene responsible for usher syndrome type 1b. *Nature.* 1995;374(6517):60–1. 10.1038/374060a0 7870171

[8] Roux AF Faugère V Le Guédard S : Survey of the frequency of ush1 gene mutations in a cohort of usher patients shows the importance of cadherin 23 and protocadherin 15 genes and establishes a detection rate of above 90%. *J Med Genet.* 2006;43(9):763–8. 10.1136/jmg.2006.041954 16679490 PMC2564578

[9] Jaijo T Aller E Beneyto M : *Myo7a* mutation screening in usher syndrome type i patients from diverse origins. *J Med Genet.* 2007;44(3):e71. 10.1136/jmg.2006.045377 17361009 PMC2598023

[10] Yoshimura H Miyagawa M Kumakawa K : Frequency of usher syndrome type 1 in deaf children by massively parallel dna sequencing. *J Hum Genet.* 2016;61(5):419–22. 10.1038/jhg.2015.168 26791358 PMC4893503

[11] Jouret G Poirsier C Spodenkiewicz M : Genetics of usher syndrome: New insights from a meta-analysis. *Otol Neurotol.* 2019;40(1):121–129. 10.1097/MAO.0000000000002054 30531642

[12] Ammar-Khodja F Faugère V Baux D : Molecular screening of deafness in algeria: high genetic heterogeneity involving dfnb1 and the usher loci, dfnb2/ush1b, dfnb12/ush1d and dfnb23/ush1f. *Eur J Med Genet.* 2009;52(4):174–9. 10.1016/j.ejmg.2009.03.018 19375528

[13] Sang Q Yan X Wang H : Identification and functional study of a new missense mutation in the motor head domain of myosin viia in a family with autosomal dominant hearing impairment (dfna11). *PLoS One.* 2013;8(1):e55178. 10.1371/journal.pone.0055178 23383098 PMC3558421

[14] Zallocchi M Meehan DT Delimont D : Role for a novel usher protein complex in hair cell synaptic maturation. *PLoS One.* 2012;7(2):e30573. 10.1371/journal.pone.0030573 22363448 PMC3281840

[15] Dallos P : The active cochlea. *J Neurosci.* 1992;12(12):4575–85. 10.1523/JNEUROSCI.12-12-04575.1992 1464757 PMC6575778

[16] Ashmore J Avan P Brownell WE : The remarkable cochlear amplifier. *Hear Res.* 2010;266(1–2):1–17. 10.1016/j.heares.2010.05.001 20541061 PMC6366996

[17] Ashmore J : Outer hair cells and electromotility. *Cold Spring Harb Perspect Med.* 2019;9(7):a033522. 10.1101/cshperspect.a033522 30181355 PMC6601450

[18] Guinan JJ Jr : Olivocochlear efferents: Their action, effects, measurement and uses, and the impact of the new conception of cochlear mechanical responses. *Hear Res.* 2018;362:38–47. 10.1016/j.heares.2017.12.012 29291948 PMC5911200

[19] Smalt CJ Heinz MG Strickland EA : Modeling the time-varying and level-dependent effects of the medial olivocochlear reflex in auditory nerve responses. *J Assoc Res Otolaryngol.* 2014;15(2):159–73. 10.1007/s10162-013-0430-z 24306278 PMC3946143

[20] Boero LE Castagna VC Di Guilmi MN : Enhancement of the medial olivocochlear system prevents hidden hearing loss. *J Neurosci.* 2018;38(34):7440–7451. 10.1523/JNEUROSCI.0363-18.2018 30030403 PMC6104299

[21] Yang Q Sun G Cao Z : The expression of nlrx1 in c57bl/6 mice cochlear hair cells: Possible relation to aging- and neomycin-induced deafness. *Neurosci Lett.* 2016;616:138–46. 10.1016/j.neulet.2015.11.053 26836140

[22] Viana LM O’Malley JT Burgess BJ : Cochlear neuropathy in human presbycusis: Confocal analysis of hidden hearing loss in post-mortem tissue. *Hear Res.* 2015;327:78–88. 10.1016/j.heares.2015.04.014 26002688 PMC4554812

[23] Hasson T Gillespie PG Garcia JA : Unconventional myosins in inner-ear sensory epithelia. *J Cell Biol.* 1997;137(6):1287–307. 10.1083/jcb.137.6.1287 9182663 PMC2132524

[24] Li S Mecca A Kim J : Myosin-viia is expressed in multiple isoforms and essential for tensioning the hair cell mechanotransduction complex. *Nat Commun.* 2020;11(1):2066. 10.1038/s41467-020-15936-z 32350269 PMC7190839

[25] Sandquist JC Means AR : The c-terminal tail region of nonmuscle myosin ii directs isoform-specific distribution in migrating cells. *Mol Biol Cell.* 2008;19(12):5156–67. 10.1091/mbc.e08-05-0533 18843042 PMC2592670

[26] Oliver TN Berg JS Cheney RE : Tails of unconventional myosins. *Cell Mol Life Sci.* 1999;56(3–4):243–57. 10.1007/s000180050426 11212352 PMC11147021

[27] Weston MD Pierce ML Jensen-Smith HC : Microrna-183 family expression in hair cell development and requirement of micrornas for hair cell maintenance and survival. *Dev Dyn.* 2011;240(4):808–19. 10.1002/dvdy.22591 21360794 PMC3072272

[28] Smeti I Savary E Capelle V : Expression of candidate markers for stem/progenitor cells in the inner ears of developing and adult gfap and nestin promoter-gfp transgenic mice. *Gene Expr Patterns.* 2011;11(1–2):22–32. 10.1016/j.gep.2010.08.008 20817025

[29] Dong Y Sui L Yamaguchi F : Phosphatase and tensin homolog deleted on chromosome 10 regulates sensory cell proliferation and differentiation of hair bundles in the mammalian cochlea. *Neuroscience.* 2010;170(4):1304–13. 10.1016/j.neuroscience.2010.08.024 20727948

[30] Sirko P Kozlov A : Immunohistochemistry localises myosin-7a to cochlear efferent boutons.Dryad, Dataset, 2021. 10.5061/dryad.9s4mw6mhz PMC8866907 35224213

[31] Sirko P Kozlov A : Immunohistochemistry localises myosin-7a to cochlear efferent boutons. *Zenodo.* 2021. 10.5281/zenodo.5763739 PMC8866907 35224213

