# Supplementary material for: Immunohistochemistry localises myosin-7a to cochlear efferent boutons
Source: Wellcome Open Res. 2022 Feb 22;7:1. Originally published 2022 Jan 4. [Version 2] doi: 10.12688/wellcomeopenres.17428.2 (PMC8866907; doi:10.12688/wellcomeopenres.17428.2)
Supplement: Peer review history

# Peer review: Reviewer response for version 2

**DOI:** 10.21956/wellcomeopenres.19617.r48849

**Published:** 2022-02-23

**Authors:** Walter Marcotti

**Affiliations:** 1 School of Biosciences, University of Sheffield, Sheffield, South Yorkshire, S10 2TN, UK

The authors have fully addressed my points.

Is the work clearly and accurately presented and does it cite the current literature?

Partly

If applicable, is the statistical analysis and its interpretation appropriate?

Not applicable

Are all the source data underlying the results available to ensure full reproducibility?

Yes

Is the study design appropriate and is the work technically sound?

Yes

Are the conclusions drawn adequately supported by the results?

Partly

Are sufficient details of methods and analysis provided to allow replication by others?

Yes

Reviewer Expertise:

Auditory Neuroscience

I confirm that I have read this submission and believe that I have an appropriate level of expertise to confirm that it is of an acceptable scientific standard.

# Peer review: Reviewer response for version 1

**DOI:** 10.21956/wellcomeopenres.19271.r47885

**Published:** 2022-01-24

**Authors:** Hiroshi Hibino

**Affiliations:** 1 Osaka University Graduate School of Medicine, Osaka, Japan

This study examined the localization of myosin-7a in the cochlea of adult rat by immunohistochemistry. This actin-binding protein is involved in the formation of hair-cell’s stereocilia. The authors detected the strong myosin-7a immunoreactivity not only in the hair-cell’s elements as reported previously but also in the medial olivocochlear efferent synaptic boutons contacting the outer hair cells. The signal was clearly observed even when the secondary antibody was changed to the different one. Moreover, minimum fluorescence was visible in the experiment without the primary myosin-7a antibody. These observations sufficiently support their idea that myosin-7a is also present in the efferent synaptic boutons.

 The quality of the data is convincing and the text is well written. To improve the manuscript, I described a few minor comments in the followings.

 Minor issues

1. For a broad array of readers including non-experts, I request the authors to describe an illustration of an organ of Corti in the manuscript. In addition, they should point to the positions of outer hair cells and inner hair cells by arrows or arrowheads in all the figures.
2. I suggest the authors to show the depth information in all the figures. For instance, information of the distance between the apical or upper surface of hair cells and the immunolabeling in the button will be helpful.
3. Describe the age of the animals examined in this study.
4. In page 6, left column, 2 ^nd^ paragraph; ‘Nterminal’ is typo?

Is the work clearly and accurately presented and does it cite the current literature?

Yes

If applicable, is the statistical analysis and its interpretation appropriate?

Yes

Are all the source data underlying the results available to ensure full reproducibility?

Yes

Is the study design appropriate and is the work technically sound?

Yes

Are the conclusions drawn adequately supported by the results?

Yes

Are sufficient details of methods and analysis provided to allow replication by others?

Yes

Reviewer Expertise:

Hearing

I confirm that I have read this submission and believe that I have an appropriate level of expertise to confirm that it is of an acceptable scientific standard.

# Response

**Published:** 2022-02-12

**Authors:** Andrei Kozlov

**Affiliations:** Imperial College London, UK

We thank our reviewer Professor Hiroshi Hibino for the time and effort he has spent to carefully evaluate our manuscript and for his helpful recommendations for improvement. We provide the reviewers’ comments (in *italics*) and our responses to each point below.

*For a broad array of readers including non-experts, I request the authors to describe an illustration of an organ of Corti in the manuscript. In addition, they should point to the positions of outer hair cells and inner hair cells by arrows or arrowheads in all the figures.*

**

 We have added an illustration of the organ of Corti and a cross-section of the cochlea showing its localization in Figure 1 in the updated version of the manuscript. We have also used arrowheads to indicate the position of inner and outer hair cells in all figures.

*I suggest the authors to show the depth information in all the figures. For instance, information of the distance between the apical or upper surface of hair cells and the immunolabeling in the button will be helpful.*

 We have added information about the depth of the bouton below the apical surface of the hair cells to the figure captions.

*Describe the age of the animals examined in this study.*

 We have added information about the age of the animals to the 2nd paragraph of the methods section.

*In page 6, left column, 2* ^*nd*^ *paragraph; ‘Nterminal’ is typo?*

 Yes, this was a typo. It has been corrected in the updated version of the manuscript.

# Peer review: Reviewer response for version 1

**DOI:** 10.21956/wellcomeopenres.19271.r48008

**Published:** 2022-01-18

**Authors:** Walter Marcotti

**Affiliations:** 1 School of Biosciences, University of Sheffield, Sheffield, South Yorkshire, S10 2TN, UK

The focus of this short manuscript is to describe the distribution of Myo7a in the rat cochlea. The experimental work is performed using a combination of immunostaining and confocal imaging. The authors claim that Myo7a is not only expressed in the sensory hair cells, as previously demonstrated, but also in the efferent terminals of the medial olivocochlear fibres contacting the OHCs. This is, in principle, an interesting finding with several potential functional implications, as stated by the authors. Although this work provides a very nice descriptive investigation, there are several aspects that need some additional consideration. This will require either additional experiments or some clear statements indicating the several limitations of this brief study.

 One of the most unclear aspects of the work is the discrepancy between some of the data provided. Figures 1 and 2 show very little, or no overlap between Myo7a and neurofilament, which should label the efferent terminals. However, using the same antibodies, the authors claim the opposite in Figure 4 (complete overlap; see also the last point below). Considering that the images should be representative of the results, I am unsure which piece of evidence I should consider. Maybe the use of an additional anti-Myo7a antibody should be sought.

 Also, have the authors considered the possibility that these round-shaped Myo7a positive structures could be part of unhealthy OHCs or something else? This could be easily addressed by double labelling the OHCs with another hair cell marker (e.g. Myo6).

 From the data provided, it is also very difficult to appreciate the possible overlap highlighted in Figure 4. How are the authors able to distinguish between overlap (Myo7a in both in the OHCs and efferent terminals) or its juxtaposition (Myo7a in OHCs and NF or VAChT in the efferent terminals) from the images provided?

Is the work clearly and accurately presented and does it cite the current literature?

Partly

If applicable, is the statistical analysis and its interpretation appropriate?

Not applicable

Are all the source data underlying the results available to ensure full reproducibility?

Yes

Is the study design appropriate and is the work technically sound?

Yes

Are the conclusions drawn adequately supported by the results?

Partly

Are sufficient details of methods and analysis provided to allow replication by others?

Yes

Reviewer Expertise:

Auditory Neuroscience

I confirm that I have read this submission and believe that I have an appropriate level of expertise to confirm that it is of an acceptable scientific standard, however I have significant reservations, as outlined above.

# Response

**Published:** 2022-02-12

**Authors:** Andrei Kozlov

**Affiliations:** Imperial College London, UK

We would like to thank our reviewer Professor Walter Marcotti for the time and effort he has spent to carefully evaluate our manuscript and for the helpful recommendations for improvement. We provide the reviewers’ comments (in *italics*) and our responses to each point below.

*One of the most unclear aspects of the work is the discrepancy between some of the data provided. Figures 1 and 2 show very little, or no overlap between Myo7a and neurofilament, which should label the efferent terminals. However, using the same antibodies, the authors claim the opposite in Figure 4 (complete overlap; see also the last point below). Considering that the images should be representative of the results, I am unsure which piece of evidence I should consider. Maybe the use of an additional anti-Myo7a antibody should be sought.*

 The results in several publications we reviewed whilst writing the manuscript were consistent with the data we obtained. Similarly to our results, neurafilament staining did not visualise well the efferent boutons; instead antibodies against other markers such as VAChT, or anti-choline acetyltransferase (ChAT) were used for this purpose. Examples can be seen in Figure1 (Froud *et. al.*, 2015: https://www.nature.com/articles/ncomms8115#MOESM736), Figure 4 (Lang *et. al.*, 2011: https://pubmed.ncbi.nlm.nih.gov/21061038/), Figure 4 (Seist et. al., 2020: https://www.frontiersin.org/articles/10.3389/fnmol.2020.00087/full) and Figure 5 (Kujawa and Liberman 2005: https://www.jneurosci.org/content/29/45/14077/tab-figures-data).

 NF-H staining visible in Figure 5 at the location of the medial olivocochlear boutons is comparable to the NF-H staining of the organ of Corti fragment shown in Figure 2. This is not clear in Figure 2 as it depicts a composite image showing both myosin 7a and NF-H staining but can be verified by viewing the freely available Dryad dataset linked to the publication.

*Also, have the authors considered the possibility that these round-shaped Myo7a positive structures could be part of unhealthy OHCs or something else? This could be easily addressed by double labelling the OHCs with another hair cell marker (e.g. Myo6).*

 We have not conducted separate double labelling experiments, however the localization of the Myo7a positive structures clearly overlaps with the localization of VAChT as shown in the orthogonal views found in Figures 6 and 7 in version 2 of the manuscript.

*From the data provided, it is also very difficult to appreciate the possible overlap highlighted in Figure 4. How are the authors able to distinguish between overlap (Myo7a in both in the OHCs and efferent terminals) or its juxtaposition (Myo7a in OHCs and NF or VAChT in the efferent terminals) from the images provided?*

 To better show the position of the round-shaped Myo7a positive structures we have created orthogonal views of the image stacks we captured (Figures 6 and 7 in the updated version of the manuscript). Views were captured along the X-axis shown on the top-down views in those figures.

 The orthogonal views appear to confirm that the round-shaped Myo7a positive structures are separate from OHCs and are located below them. Furthermore, the orthogonal views confirm the colocalisation of these structures with the efferent bouton marker VAChT.
